# An insertion mutation of the MECP2 gene in severe neonatal encephalopathy and ocular and oropharyngeal dyskinesia: a case report

**DOI:** 10.1186/s12920-023-01616-6

**Published:** 2023-08-03

**Authors:** Jianmin Liang, Cuijuan Xin, Meiying Xin, Guangliang Wang, Xuemei Wu

**Affiliations:** 1 Department of Pediatric, Neurology of Jilin University, 1 Xinmin Street, Changchun, 130000 Jilin Province China; 2 Jilin Provincial Key Laboratory of Pediatric Neurology, Changchun, China; 3 Department of Cardiology, Jiren Hospital of Far Eastern Horizon, Anda, China

**Keywords:** MECP2, Ocular dyskinesia, Oropharyngeal dyskinesia, Severe neonatal encephalopathy, Insertion mutation

## Abstract

**Background:**

Pathogenic variation of the MECP2 gene presents mostly as Rett syndrome in females and is extremely rare in males. Most male patients with MECP2 gene mutation show MECP2 duplication syndrome.

**Case presentation:**

Here we report a rare case in a 10-month-old boy with a hemizygous insertion mutation in *MECP2* as NM_001110792, c.799_c.800insAGGAAGC, which results in a frameshift mutation (p.R267fs*6). The patient presented with severe encephalopathy in the neonatal period, accompanied by severe development backwardness, hypotonia, and ocular and oropharyngeal dyskinesia. This is the first report of this mutation, which highlights the phenotype variability associated with MECP2 variants.

**Conclusions:**

This case helps to expand the clinical spectrum associated with *MECP2* variants. Close attention should be paid to the growth and development of patients carrying a *MECP2* variant or Xq28 duplication. Early interventions may help improve symptoms to some certain extent.

## Background

The methyl CpG binding protein 2 (MECP2) gene (OMIM 300,005) located on the X chromosome encodes an important regulator of brain development and is a key dose-sensitive gene in neuronal development that needs to be expressed at precise levels in cells [1]. Aberrations or variations in the MECP2 gene lead to partial or total loss of protein function, resulting in Rett syndrome (RTT) (OMIM 312,750), a rare disease in females with an incidence rate of approximately 1/15,000. RTT mainly manifests as developmental regression, seizures and repetitive stereotyped hand movements [2, 3]. Pathogenic variations of the MECP2 gene in males are extremely rare and usually lead to embryonic death. The clinical manifestations of male patients with *MECP2* variations are diverse, ranging from mild non-specific mental retardation to severe neonatal encephalopathy [3, 4].

Here we report a rare case in a 10-month-old boy with an insertion mutation in the MECP2 gene, NM_001110792(MECP2): c.799_800insAGGAAGC, which causes a frameshift mutation (p.R267fs*6). The clinical phenotype of the patient was severe encephalopathy in the neonatal period, accompanied by severe developmental delay, hypotonia, and ocular and oropharyngeal dyskinesia, which has not been reported before. This case expands the clinical presentations of MECP2 gene mutations, particularly in males, that may help improve early identification and intervention.

## Case presentation

The proband was a 10-month-old boy who was admitted because of ocular and oropharyngeal dyskinesia for 1 day. The patient had apnea for 15 min after birth and was diagnosed with “neonatal asphyxia, meconium aspiration syndrome, neonatal pneumonia, respiratory failure, hemolytic disease of the newborn, and atrial septal defect.” He was given Continuous Positive Airway Pressure as an auxiliary treatment and anti-infection treatment for 7 days. The child’s condition improved.

**Fig. 1:**
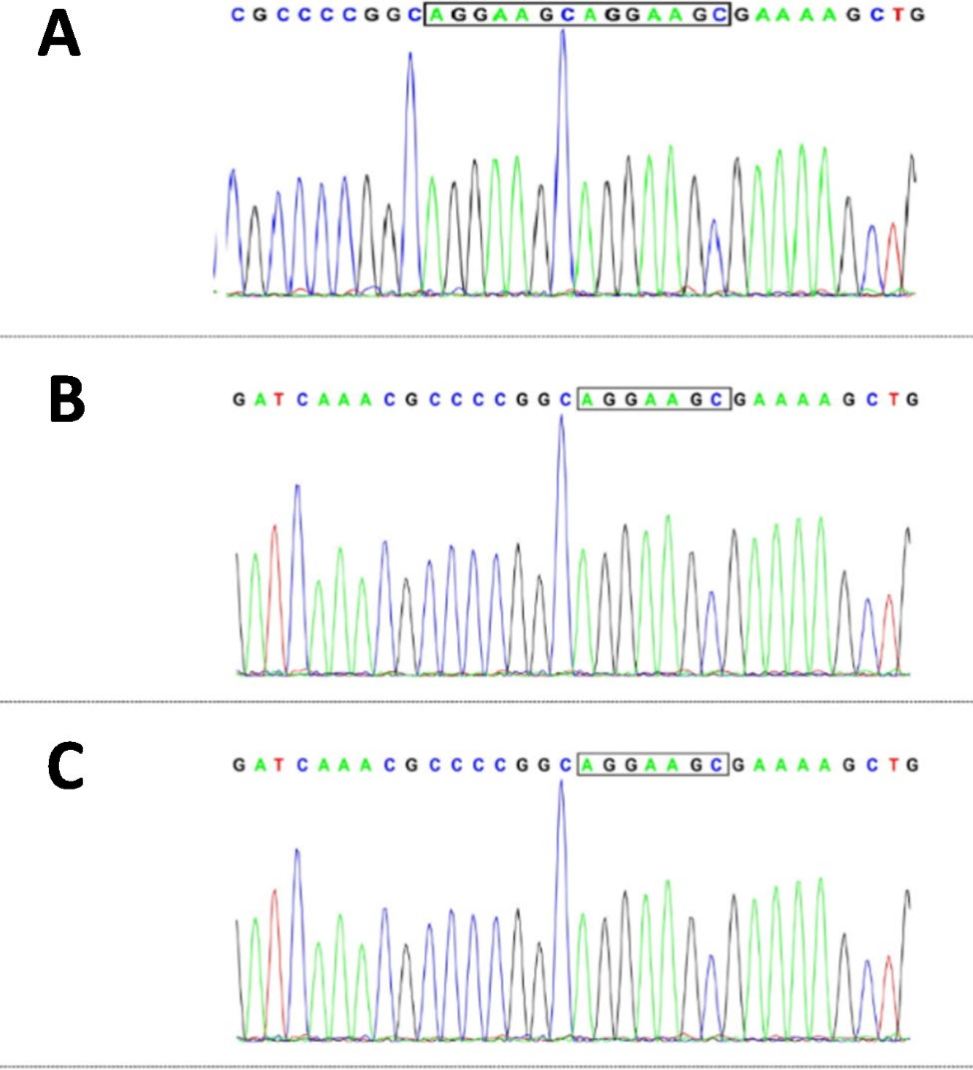
MECP2 gene sequencing results. chrX: 153,296,522,153,296,523; NM_001110792: c.799_800insAGGAAGC; p.R267fs*6(p.Arg267fsTer6). A de novo mutation was identified in the MECP2 gene of the patient (A). The mutation was not found in DNA samples from the patient’s father (B) and mother (C)

The baby was delivered at 39 weeks of gestation by Cesarean section because of the mother’s placenta hypofunction; the baby’s weight was 3.89 kg at birth. Apgar score was 7 points for 1 min and 8 points for 5 and 10 min after birth. Both parents are healthy. The developmental milestones of the patient were normal. Findings from physical examination followed up at 10 months were as follows: weight 7.7 kg, height 70 cm, tracking parallax, paroxysmal binocular vision, paroxysmal oropharyngeal involuntary movement, hypotonia, and no abnormality in lung and abdomen. Routine laboratory testing revealed normal complete blood counts, and the liver aspartase, blood urea nitrogen, creatinine, and electrolyte levels were all within normal ranges. EEG showed abnormal waveforms with spike slow waves and slow waves in the bilateral frontal, central, parietal and midline areas. Head MRI showed mild brain atrophy. With the informed consent of the proband’s parents, 2 ml of the proband’s venous blood and his father’s and mother’s venous blood was collected in EDTA anticoagulant tubes for sequencing. The MECP2 gene in the parents was wild-type, and the child had a de novo mutation in the MECP2 gene (NM_001110792(MECP2): c.799_800insAGGAAGC), which causes a frameshift mutation (p.R267fs*6) (Fig. 1).

Following the diagnosis of severe neonatal encephalopathy phenotype caused by MECP2 gene mutation (c.799_800insAGGAAGC). Movement and intelligence rehabilitations were prescribed. However, the patient has not shown much improvement.

## Discussion and conclusions

Here we report a rare case of a male child with severe neonatal encephalopathy caused by a frameshift mutation in exon 3 of the MECP2 gene, NM_001110792: c.799_800insAGGAAGC, which has not been reported. The clinical phenotype of severe neonatal encephalopathy with ocular and oropharyngeal dyskinesia has also not been reported.

MECP2-related disorders in males are present in 1.7% of male patients with intellectual disability [1]. Few reports have been published on males with neonatal encephalopathy and/or Rett syndrome with a variant in the MECP2 gene in the absence of mosaicism or presence of extra X chromosome. RTT is a X-linked dominant inheritance. Methylated cytosine deamination occurs in rapidly dividing germ cells (sperm), and most of them are de novo mutations [1, 2]. RTT almost always occurs in women, and it is extremely rare for men to develop RTT. The clinical variation of *MECP2* mutations in males is significant, making these patients more prone to severe neurodevelopmental problems such as initial developmental limitations, increased ventilation demand, and early death [4]. These clinical features are not the same as those observed in female RTT cases and can be used for general diagnosis of male RTT.

The phenotypes of MECP2-related disorders in males are highly heterogeneous (Table 1). MECP2-related disorders in males can occur in RS cases. These patients exhibit severe neonatal encephalopathy and usually die in the first year of life. When these mutations occur in normally expressed cells, it can lead to a milder phenotype (and clinical RS). This is the case with XXY boys. The mutation in patients not found in women with RS are usually compatible with life in adulthood. The neurological manifestations range from severe to mild non-specific MR. The main clinical features of males associated with MECP2 duplication syndrome are non-specific, but they include severe phenotypes. The phenotype of *MECP2* mutations in males can be divided into three categories: (1) severe neonatal encephalopathy (OMIM# 300,673), which involves the same variation as typical female patients with Rett syndrome; these patients show severe neonatal encephalopathy, respiratory failure, low muscular tension, lower limb stiffness, dyskinesia, seizures without structural abnormalities of the brain, and even early death in children [5, 6]; (2) *MECP2* mutation with a typical RTT phenotype [7, 8]; and (3) X-linked mental retardation syndrome type 13 (OMIM# 300,055), a rare missense mutation in females with typical RTT, which shows atypical features such as mental retardation, mental disorder or autism spectrum disorder [9]. MECP2 gene duplications in males present as MECP2 duplication syndrome (OMIM #300,260) (3). The genotype–phenotype correlations of these patients have been difficult to determine because of the small number of reported cases. The phenotype of the proband should be classified as severe neonatal encephalopathy (OMIM# 300,673).

**Table 1:** Mutation type and phenotype-genotype correlations in male MECP2 patients

Genotype		Phenotype
Mutations in MECP2	Normal chromosomal complement (46, XY) The same mutations cause RTT in girls	Severe neonatal encephalopathy (OMIM #300,673)
	Mosaic or Klinefelter syndrome (47, XXY) The same mutations cause RTT in girls	Classic or atypical Rett syndrome (RTT) (OMIM #312,750)
	Normal chromosomal complement (46, XY) The same mutations do not affect girls	X-linked mental retardation syndrome type 13 (OMIM #300,055)
Duplications of MECP2	Whole gene duplication of the MECP2	MECP2 duplication syndrome (MDS) (OMIM #300,260)

Genotype–phenotype correlations have been difficult to determine because of the low number of reported cases

Genetic sequencing in the current case revealed a hemizygous frameshift mutation in exon 3 of the MECP2 gene (c.799_800insAGGAAGC), which results in a frameshift mutation (p.R267fs*6). The parents showed no mutation at this site, and thus this is a de novo mutation. The minor allele frequency is extremely low in databases of genomAD. Following the American College of Medical Genetics and Genomics (ACMG) guidelines, c.799_800insAGGAAGC, which causes p.R267fs*6, a frameshift mutation, was determined to be pathogenic (PVS1 + PM2 + PM6). The neonatal behavioral neurological assessment score of the current case is only 19 points. The patient showed asphyxia, meconium aspiration syndrome, neonatal pneumonia, respiratory failure, hemolytic disease, atrial septal defect, and severe encephalopathy in the neonatal period. In addition, abnormal muscular tension, multifocal epileptiform waves of EEG, and mild atrophy of bilateral cerebral hemispheres of the cranial nucleus and magnetic field were observed, which are consistent with the early clinical characteristics of male severe neonatal encephalopathy phenotype caused by MECP2 gene mutation. The rare ocular and oropharyngeal dyskinesia in the 10-month-old boy may be caused by dyskinesia from the involvement of basal ganglia. There is no specific treatment for this disease. Early interventions, such as rehabilitations, may help improve symptoms to a certain extent. Studies have suggested that gene editing technology may be the best choice to significantly improve disease symptoms or to cure this disease, and research in this area has shown promising findings [10].

The MECP2 gene is located on chromosome Xq28. Over 800 different *MECP2* mutations have been found in more than 95% of classic RTT patients and 75% of atypical RTT patients [1]. *MECP2* has several hotspot mutations, including R106W, R133C, T158M, R168X, R255X, R270X, R294X, and R306C, which account for 70% of the total mutations. Mutations at the C-terminus are mainly small fragment deletions, accounting for approximately 8% of all mutations, and large fragment deletions, accounting for approximately 5% of all mutations [1, 2].

In conclusion, here we report the first case of NM_001110792: c.799_800insAGGAAGC with p.R267fs*6, an insertion in the MECP2 gene, in a male patient who presented with severe neonatal encephalopathy in the early stage, followed by ocular and oropharyngeal dyskinesia at the age of 10 months. There is a wide spectrum of clinical presentation associated with *MECP2* variants that is difficult to diagnose. Therefore, clinical attention should be paid to all male patients carrying a *MECP2* variant or a Xq28 duplication. Early family intervention should be encouraged, and regular follow-up should be carried out.

## Data Availability

All data generated or analysed during this study are included in this published article.

## List of abbreviations

MECP2: Methyl CpG binding protein 2
RTT: Rett syndrome
CPAP: Continuous positive airway pressure
ACMG: American College of Medical Genetics and Genomics
ExAC: Exome Aggregation Consortium
gnomAD: Genome Aggregation Database
PVS: Pathogenic very strong
PM: Pathogenic moderate
EEG: Electro-encephalogram
NGS: Next generation sequencing
